# Scalable Data Model for Traffic Congestion Avoidance in a Vehicle to Cloud Infrastructure

**DOI:** 10.3390/s21155074

**Published:** 2021-07-27

**Authors:** Ioan Stan, Vasile Suciu, Rodica Potolea

**Affiliations:** Department of Computer Science, Technical University of Cluj-Napoca, 26-28 G. Baritiu, 400027 Cluj-Napoca, Romania; vasile.suciu@student.utcluj.ro (V.S.); rodica.potolea@cs.utcluj.ro (R.P.)

**Keywords:** connected vehicles, scalability, data structures, congestion avoidance, urban traffic, simulation

## Abstract

Traffic congestion experience in urban areas has negative impact on our daily lives by consuming our time and resources. Intelligent Transportation Systems can provide the necessary infrastructure to mitigate such challenges. In this paper, we propose a novel and scalable solution to model, store and control traffic data based on range query data structures (K-ary Interval Tree and K-ary Entry Point Tree) which allows data representation and handling in a way that better predicts and avoids traffic congestion in urban areas. Our experiments, validation scenarios, performance measurements and solution assessment were done on Brooklyn, New York traffic congestion simulation scenario and shown the validity, reliability, performance and scalability of the proposed solution in terms of time spent in traffic, run-time and memory usage. The experiments on the proposed data structures simulated up to 10,000 vehicles having microseconds time to access traffic information and below 1.5 s for congestion free route generation in complex scenarios. To the best of our knowledge, this is the first scalable approach that can be used to predict urban traffic and avoid congestion through range query data structure traffic modelling.

## 1. Introduction

Traffic congestion is one of the major challenges encountered by our world, especially in the urban areas. Because of this, drivers spend a lot of their time in traffic: billions of hours of extra time sitting in traffic which results in hundreds of billions of USD congestion cost [1,2]. In the major US urban areas 32% of the daily travel time occurred under congested traffic [3].

Intelligent Transportation Systems (ITS) come with approaches that attempt to predict traffic and dynamically enhance vehicle routes to avoid congestion by correlating knowledge from a large spectrum of data. Such amount of data also comes with specific challenges such as scalability [4]. A key aspect of ITS that impacts scalability is the information sharing between vehicles. Clear overviews of the state-of-the-art in the ITS development and vehicular communication are done in [5,6] while more specific information sharing approaches that use various communication channels and architectures are presented in [7,8,9]. As shown in [9], besides the specific features supported by different architectures of the traffic applications, the performance of such applications can be influenced by their architecture: centralized vs. decentralized. ITS’ traffic prediction and congestion avoidance is strongly related to route planning algorithms. Route planning algorithms use and impact different aspects (e.g., traffic information) of daily travel experience as is presented and evaluated in various works [10,11,12,13,14,15,16]. From implementation cost perspective, it was shown that traffic modeling through simulation is an efficient and reliable approach [17,18,19,20,21,22,23]. Considering all the above mentioned challenges and achievements, we found that there is an urgent need to develop a scalable traffic modeling database that can be used by navigation systems to predict and avoid congestion based on accurate traffic information during route planning.

The main goal of this work is to develop a novel and scalable traffic modeling solution in order to efficiently predict and avoid traffic congestion in urban areas. The main contributions of this paper are:

1. We present a conceptual architecture of the traffic congestion prediction strategy based on three main pillars in order to support traffic storage and traffic control: Map Topology, Route Planning Algorithms, Range Query Data Structures. Similar to the methodology in [24], our work uses route planning algorithms to generate routes considering the map topology and the existing real-time information about traffic (already generated routes) that are efficiently stored using range query data structures.
2. Two novel range query data structures K-ary Interval Tree and K-ary Entry Point Tree) that can be used to represent and control large-scale traffic information in a V2C infrastructure.
3. The KI Tree and KEP Tree were integrated with the OSMAnd Navigation System [25] in order to store and control the generated vehicle routes.
4. Adaptation of the OSMAnd Navigation System to behave like a cloud service. OSMAnd’s Route Planning Algorithm was adapted to support tens thousands of concurrent routes on the road network (map) in order to simulate traffic in urban areas.

The rest of the paper is structured as follows. The Section 2 discusses related work from the literature. In Section 3 we present the conceptual architecture and pillars of the used traffic congestion prediction and avoidance strategy. In the Section 4 we present and analyze the conceptual model of the traffic database from data structure perspective. Section 5 contains a description of the algorithms used to efficiently process and store traffic in the V2C infrastructure database. The evaluation and experimental results of the proposed solution are discussed in Section 6. The Section 7 concludes the paper and gives an overview of the planned future work.

## 2. Related Work

Different aspects of the vehicular traffic were discussed, analyzed and evaluated by the literature in recent years. In the first two parts of this section, we present the related work in the literature regarding traffic prediction and congestion avoidance. The last subsection discusses the main known large-scale traffic simulation solutions from the literature.

### 2.1. Traffic Prediction

Several traffic prediction solutions in the literature use simulation and data mining for short-term traffic prediction in non-urban areas. One worth-mentioning solution that predicts the fundamental traffic parameters speed, flow and density [26,27] is proposed in [28] and is based on online change-point-based (OCPB) model. The work in [29] describes a short-term traffic flow prediction approach based on dynamic tensor completion (DTC). An interpretable and adaptable spatio-temporal Bayesian multivariate adaptive-regression splines (ST-BMARS) approach for short-term highway traffic prediction is described in [30] and is shown its superiority in comparison with temporal multivariate adaptive regression splines (MARS) model, the parametric auto regressive integrated moving average (ARIMA) model, the state-of-the-art seasonal ARIMA model and the kernel method support vector regression. Another perspective for predicting short-term traffic flow is presented in [31] and is based on a unified spatio-temporal model. Its behaviour depends on the road network topology. The authors of this proposal proved that its accuracy is superior to space-time auto regressive integrated moving average (STARIMA) and back propagation neural network (BPNN) approaches on freeway traffic prediction. Spatio-temporal related approaches for short-term traffic flow prediction can be found also in [32,33]. The authors in [33] use a space-time k-nearest neighbour (ST-kNN) method to predict highway short-term traffic. Deep learning approaches to predict traffic information are presented in [34,35]. The work in [35] uses weather information to predict traffic flow on highways while the approach in [34] tries to predict short-term traffic based on traffic data from ring roads in Beijing. The work in [17,36,37] are simulation-based traffic prediction approaches. In [36] are used Generalized Beta-Gaussian Bayesian Networks on less than 250 map links while in [37] is used SUMO [38] to simulate traffic in Cologne, Germany for two models that predicts traffic on time intervals that are less than 1 min and greater than 1 min, respectively. A macroscopic traffic flow model is used in [3] to real-time traffic prediction and congestion on highways. The work in [3] is able to warn the driver in less than 7 s before entering traffic jam. Highway traffic prediction methods are also presented in [39] and are based on time-aware multivariate nearest neighbour regression algorithms. A segment-based regression kriging (SRK) method is presented in [40]. It predicts traffic by differentiating heavy and light vehicles and shows that the impact of heavy vehicles on road maintenance is much larger than the one of light vehicles and it varies across space.

The literature work regarding urban traffic prediction is not that various and complete as for non-urban case. The work in [41] approaches various urban traffic indicators (e.g., flow, speed, accident risk) for prediction based on deep learning. In [42] is described a neighbor-regularized and context-aware non-negative tensor factorization model (NR-cNTF) to discover and interpret urban dynamics based on urban heterogeneous data. In this work, a large amount of historical data was processed (six million trips from 20 thousand taxis and 400 thousand POI records in Beijing) with the risk of becoming irrelevant due to fast changes of the traffic context in time. The authors of work in [43] present a STARIMA based approach that efficiently predicts travel time using large volumes of traffic data information in Berlin and Thessaloniki. A communications-oriented perspective on traffic management systems for smart cities is discussed in [44] with main focus on short-term traffic forecasting. The work in [45,46] elaborates neural network-based traffic forecasting models in urban areas.

### 2.2. Traffic Congestion Prediction and Avoidance

On top of traffic prediction approaches discussed in the previous subsection, in this section are presented various approaches for traffic congestion prediction and avoidance solutions that can reduce the time spent in traffic. The work in [47] uses unsupervised incremental learning approach for road traffic congestion detection and profiling, dynamically over time. They are evaluating 190 million vehicular movement records obtained from Bluetooth identifiers placed at the intersections in the State of Victoria, Australia, in order to predict short-term traffic. Additionally, short-term traffic congestion prediction was approached in [48] by developing a deep autoencoder neural network that was trained to learn temporal correlations of a transportation network and to predict traffic congestion on data sets from a Seattle area. An interesting and promising approach used to analyze traffic congestion is proposed by the authors in [49]. Their methodology geocodes traffic-related events that are coming from Twitter to gather training dataset on which applied a Support Vector Machine method is applied to obtain a prediction model. From this model is produced a spatio-temporal traffic information that can be used to analyze the traffic congestion in Mexico City. The authors in [50] proposed different re-routing methods to avoid traffic congestion. They use 1000 vehicles simulation (based on SUMO [38] and TraCI [51]) to test the approaches. The work in [52] presents multi-platooning leaders positioning and cooperative behavior algorithms for communicant automated vehicles to increase traffic capacity. Traffic Congestion Avoidance in Urban Areas based on Inter-Vehicular Communication is approached in [53,54]. For the work in [53] traffic simulations and measurements are done on New York map while the work in [54] is evaluated in the city of Colima, Mexico by combining inter-vehicular communications, fixed roadside infrastructure, infrastructure-to-infrastructure connectivity and big data. A traffic congestion avoidance method based on multiple agents vehicle re-routing is described in [55]. It tries to achieve a trade-off between the individual and global benefits by giving the vehicles optimal guidance suggestions to bypass a blocked road ahead. The approach was tested using artificial grid maps. In the context of microscopic traffic models, it is worth mentioning the work in [56] that approaches traffic patterns detection. Human factors impact on traffic are evaluated in [57] and proved that they have a big impact on traffic stability and can lead to sudden traffic breakdowns. A more targeted approach used to improve urban traffic and reduce congestion is the work in [58]. The proposed solution tries to optimize the lighting systems at the road network intersections by applying the Swarm Intelligence Algorithm which considers the average delay time of vehicles, the average number of stops of the vehicles and the traffic capacity as the evaluation indexes.

In addition to many works that analyzed and showed the benefits of urban traffic simulation based on TraffSim [59,60,61,62], an interesting approach for urban traffic prediction and congestion avoidance based on routing algorithms is described in [63]. They use microscopic traffic simulation of 2000 and 3000 vehicles on artificial and real world maps. This work resembles with the current work in some aspects, especially on using routing algorithms to predict and avoid congestion and on simulation step to evaluate the approach. Another route centric approach is the solution shown in [64]. This approach does not consider the overall traffic state but can re-route an individual vehicle in order to balance the traffic. The authors of [65] showed the benefits of the cooperative route planning against egoistic driving mode. Cooperative route planning is also one of the main pillars of our work.

### 2.3. Large Scale Traffic Simulation

In the previous subsection, we discussed different traffic congestion avoidance solutions, especially for urban-areas. All of the presented solutions lack one important aspect: scalability. In this subsection are discussed large-scale traffic simulation methods that were developed in past. Traffic simulation in urban areas must consider scalability in order to be able to be as close as possible to real world scenarios. Table 1 summarizes the existing solutions used to model large scale traffic scenarios in various cities. The work in [18] represents a Vehicular Network Simulator (VNS) that integrates the DIVERT 2.0 traffic simulator to mimic traffic. They were using Quad Tree data structure to represent vehicles (by their position) on the large-scale road network (Porto City). In [19,20] is integrated the INTERGRATION traffic simulator in order to accurately model large-scale traffic in Downtown LA. The vehicle storage data structure representation is not described. The authors in [21] propose a large-scale traffic modeling solution that is based on INTEGRATION traffic simulator where the vehicle positions are stored, updated and queried using Grid Cells data structure with update index. Their query operation is linear with the number of vehicles on the road network and the update operation is constant. The query operation can become a bottleneck when the query frequency is high or the number of vehicles on the road network is high (traffic congestion). An innovative traffic modeling solution is described in [22] where the traffic state is defined by the generated vehicle routes that simulates the traffic. For traffic simulation they integrated and adapted OSMAnd navigation application to generate vehicle routes like in real world route planning context. In their proposed solution, it is possible to query the number of vehicles on a road segment at a specific time, therefore also having a vehicle level accuracy. The data structure used to store, update and query traffic information is Segment Tree. As is shown in Section 6, this data structure has scaling limitations due to performance of update and query operations. Following the same methodology [24] like the approach in [22] our work is proposing a novel and scalable solution based on K-ary Tree. Using this approach, we efficiently model simulated traffic in order to predict and avoid traffic congestion in urban areas. Our proposal adapts OSMAnd navigation system to behave like a cloud service in order to simulate as much as possible real world navigation scenarios in a V2C infrastructure and at the same time to be cost effective.

## 3. Congestion Prediction Pillars

In our Vehicle to Cloud (V2C) simulated infrastructure, the entire traffic information is stored and processed by the cloud service that is represented by the adapted OSMAnd navigation application. In our solution we considered 3 main pillars that are the basis of an efficient traffic prediction and congestion avoidance solution: map topology of urban areas, data structures used to store traffic information and routing algorithms used to generate all routes in a V2C ecosystem. The congestion prediction and avoidance method applied, assigns a utility score (cost) to each road segment on the map. If a segment has the potential to become congested, its utility score is decreased, making it less likely to be picked by the routing algorithm in favor of other alternative road segments (that become alternatives). In this way, the traffic is spread over a larger part of the road network in an attempt to minimize traffic congestion.

**Figure 1:**
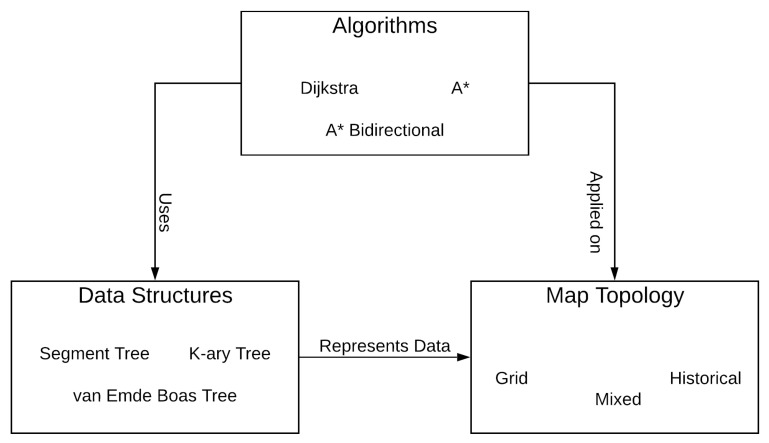
Vehicle to Cloud Congestion Prediction Pillars.

In Figure 1 are represented the 3 pillars and their relations. Considering the pillars’ classification in [24], for different map topologies (e.g., grid, historical, mixed) can be used different range query data structures (e.g., Segment Tree, K-ary Interval Tree, K-ary Entry Point Tree, van Emde Boas Tree) to store the traffic information on road segments on the map. On top of this, different route generation algorithms (e.g., A*, A* Bidirectional, Dijkstra) can be used to generate vehicle routes based on map data and traffic information. Moreover, there can be scenarios where it is more efficient to apply a specific route planning algorithm over a specific map topology [16].

It is worth mentioning that the key element that provides the ability to have an overall perspective and control over traffic distribution on roads, is the data representation and storage strategy of the computed routes that generate the traffic data. The most granular level for data representation in our proposal is based on the road segment. In our case, a road segment is a map element that represents a unique portion of a road. A road is composed by multiple road segments. For simplification, in the rest of the paper, we refer to a road segment using the term segment. Based on this data representation approach we propose a cost effective and efficient solution that uses simulation to generate random routes that are supposed to be followed by the vehicles. Considering all the above, for our purpose we adapted OSMAnd navigation system to be used as a cloud service that store and control traffic information in a range query data structure through route planning algorithm. We modified OSMAnd navigation solution to be able to request tens of thousands of concurrent routes generation in a short amount of time. OSMAnd is a mobile navigation solution on Android that uses Open Street Map (OSM) that contains all the necessary information for vehicle navigation (roads, POIs, speed limits, traffic light information, etc.). In this way, the simulation environment is close to reality from map data and route planning perspective.

The route planning algorithm from OSMAnd application was modified to consider and store (through range query data structures) already generated traffic and potential traffic congestion generation when a segment is used to compute a route. Algorithm 1 shows the simplified flow of the route planning algorithm we used. It is a modified version of the bidirectional A* route search algorithm where the number of backward steps is limited to a constant c. In our case c = 10 and ensures that the destination point is reached. The value was found after multiple evaluations of the algorithm during testing.
**Algorithm 1** Forward Oriented Search A* Algorithm1:**procedure**Computr Route(*p_s_, p_d_*)2:    $init(forwardQueue,p_{s})$▹ initialize forward graph search cost queue3:    $init(backwardQueue,p_{d})$▹ initialize backward graph search cost queue4:    backwardSteps←105:    **while** forward search unmet all backward processed segments **do**6:        forwardHead←forwardQueue.head()7:        backwardHead←backwardQueue.head()8:        **if** backwardSteps==0 OR forwardHead<backwardHead **then**9:           segmentID←forwardQueue.pop()10:        **else**11:           segmentID←backward.pop()12:           backwardSteps←backwardSteps−113:        **end if**14:        processSegment(segmentID)15:    **end while**16:**end procedure**

The key step of the algorithm used to predict and avoidance congestion is the processing segment statement that is presented in Algorithm 2. The notations used by Algorithm 2 are defined as follows:

- p_s_—starting point of a route given as GPS coordinates
- p_d_—destination point of a route given as GPS coordinates
- segmentID—segment on the navigation map represented by segment ID
- C_map_(segmentID)—map cost value of a segment from the map (e.g., Euclidean distance, turn costs, speed limit on the segment, etc.)
- N(segmentID)—set of neighbour segments of segmentID
- visited(segmentID)—returns TRUE if segmentID was visited in the routing algorithm search graph and FALSE otherwise
- C_max_—constant representing the maximum cost value possible on the map (used to limit the forward search exploration algorithm)
- C_vehicles_(ρ(segment,t))—cost factor corresponding to predicted vehicles’ density on a segment at a specific time
- θ—threshold value that indicates traffic congestion
- vehiclesCount(segmentID)—total number of connected vehicles that navigates through a specific segment on the map
- vehicleLength—the average length of a vehicle. In this work we considered to be 7 meters
- lanesCount(segmentID)—number of lanes on a segment used for vehicles’ density computation
- predicted vehicles’ density on a segment at a specific time ρ(>segmentID,t) defined as
  $$\frac{\mathrm{vehiclesCount}(\mathrm{segmentID},t)\times \mathrm{vehicleLength}}{\mathrm{length}(\mathrm{segmentID})\times \mathrm{lanesCount}(\mathrm{segmentID})}$$

The predicted time (t) represents the entrance moment on the given segment and depends on the vehicle speed and vehicles’ density (ρ) on the roads. In the segment processing step, a segment can be considered a good candidate of becoming congested in future if the vehicles’ density on the segment reaches a threshold value θ. The algorithm addresses the congestion issue by setting the cost on the current segment to a maximum value. In this way the congestion on the current segment is predicted and, if there exist other alternative segments they are preferred by the routing algorithm and congestion is avoided on the current segment. In order to have a realistic setup of the algorithm we used for θ the value found by the work in [23].
**Algorithm 2** Process Segment1:**procedure**Process Segment(*segment*)2:    **for each** segmentID in N(segment) **do**3:        **if** [notvisited(segmentID) **then**4:           $C_{map}\leftarrow C_{map}\left(segmentID\right)$5:           t←predicted time when the vehicle arrives on segment6:           **if** θ<ρ(segmentID,t) **then**7:               $C_{vehicles}\leftarrow C_{max}$ ▹ force to try another segment8:           **else**9:               $C_{vehicles}\leftarrow C_{vehicles}\left(\rho \left(segmentID,t\right)\right)$10:           **end if**11:           $costValue\leftarrow C_{map}\times C_{vehicles}$12:           updateCost(segmentID,costValue)13:        **end if**14:    **end for**15:**end procedure**

## 4. Range Query Data Structures

The core of our system is the traffic data representation on segments. In this paper we propose a novel and scalable solution that stores and controls the traffic information in a V2C ecosystem. Our solution is basically using range query data structures as is described in this section. First of all, it is worth mentioning that range query data structures can be used to answer various traffic queries. In [24] are listed 3 queries that provide traffic information from different perspectives. The first query, Q1, focuses on the number of vehicles that pass a road during any specific time interval. The work in [22] adapts the segment tree data structure to answer this query.

Considering that Q1 should be answered during a fixed period of time (e.g., a day) and it refers to specific time intervals to be queried, we can state that a static range query data structure can be used for the purpose of answering Q1. This data structure should support efficient update operations too. Segment tree is such a data structure and it was proposed by the work in [22] for the purpose of representing traffic information. In their proposal the segment tree represents the entire time interval (e.g., a day) for traffic query and update on a specific map segment. Deeper technical description of Segment Tree can be found in [66].

In order to answer the other two traffic related queries from [24] regarding Q2: maximum number of vehicles on a road during any specific time interval and Q3: number of vehicles on a road at any specific moment, we propose, implement and analyze two novel range query data structures that store traffic information corresponding to a time interval (e.g., a day) on a specific map segment. The static property of these structures is a requirement that ensures the fact that the entire storage is used efficiently for each map segment i.e., the traffic information stored for each segment corresponds to the entire time interval we want to measure (e.g., a day).

**Figure 2:**
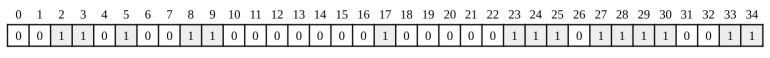
Bit Vector Representation.

**Figure 3:**
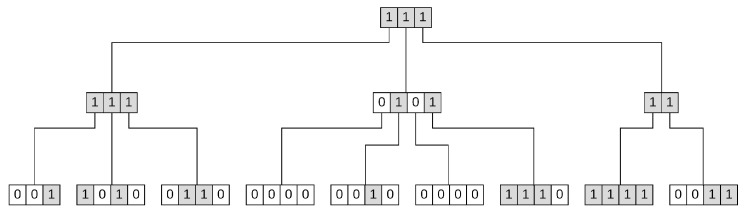
K-ary Hierarchical Bit Vector Representing 2, 3, 5, 8, 9, 17, 23, 24, 25, 27, 28, 29, 30, 33, 34.

The idea behind highway hierarchies [67] of having an overview of a certain map area and only requesting more detailed information as we need it (e.g.,we are close to required location), can be applied to the data structures as well. We can observe that this idea is well reproduced by the aggregation property of a segment tree node in [22] that aggregates traffic data from its children. There are many other tree like data structures that are based on this idea. For instance, k-ary hierarchical bit vector [68] is based on the same idea of having its nodes aggregating required information from their children. The bit vector from Figure 2 represents the set of numbers 2, 3, 5, 8, 9, 17, 23, 24, 25, 27, 28, 29, 30, 33, 34 (have the value 1 if a number is in the set, otherwise 0). This bit vector is logically represented by the k-ary hierarchical bit vector from Figure 3. The lowest level represents a single element, while the above layers aggregate information about more positions. A value of 1 at an upper layer means that there is at least one element present at a leaf rooted at the current node. From storage perspective it requires additional memory compared to the bit vector.

To answer Q2 and Q3 queries and starting from the perspective of the k-ary hierarchical bit vector, we propose two corresponding range query data structures that support traffic data representation and storage efficiently.

**Figure 4:**
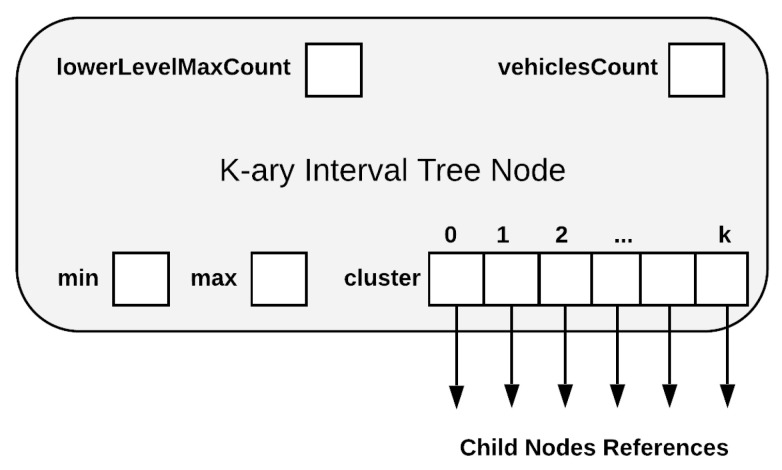
KI Tree Node Structure.

For addressing the challenges associated with Q2, we designed a K-ary Interval (KI) Tree data structure. The conceptual representation of the KI Tree Node is shown in Figure 4. KI tree node structure is a specialized K-ary tree node with the following content.

- min—represents the start time stamp of a time interval covered by the node
- max—represents the end time stamp of a time interval covered by the node
- cluster—an array of references to child nodes
- vehiclesCount—aggregates vehicle counting from its children in order to compute the maximum number of vehicles on a road during any specific time interval
- lowerLevelMaxCount—aggregates maximum vehicle counting information from its children in order to compute the maximum number of vehicles on a road during any specific time interval

The data aggregation procedure is described and exemplified in the next section together with update and query operations.

**Figure 5:**
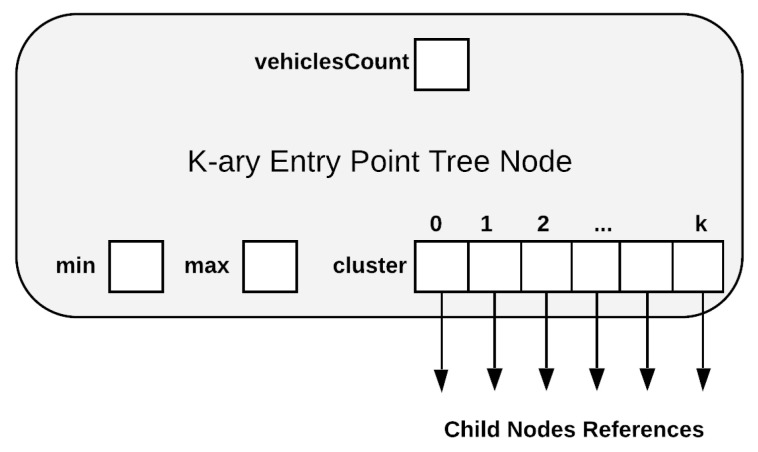
KEP Tree Node Structure.

For Q3, the specific moment is the time when a vehicle enters on a segment (entry point). For this purpose we designed a K-ary Entry Point (KEP) Tree data structure used to count the number of vehicles that enters on a segment. The logical representation of the KEP Tree Node is shown in Figure 5. The structure is similar with the KI tree node with the difference that it has only one field vehiclesCount that aggregates data from its children in order to compute the number of vehicles on a segment entrance. As for KI tree node, the data aggregation procedure is described and exemplified in the next section, together with update and query operations.

Another data model that has similar structure with ours is van Emde Boas Tree [69,70,71]. A van Emde Boas Tree node has the number of children exactly equal with the square root of maximum number of elements that can be stored in the entire tree. Together with the above discussed data structures, it is one of the range query data structures that has potential to be used to answer traffic related queries. Table 2 shows asymptotic time and space complexities for all mentioned data structures. We denote by n the number of nodes in the tree. Thee root corresponds to the entire time interval to be covered, while each node covers recursively sub-intervals of the parent node’s interval, with each second represented by a leaf in the tree. In this way n corresponds asymptotically to the number of seconds in the time interval that is covered by the root of the tree (e.g., seconds in a day).

From the experimental analysis in Section 6, we concluded that it is efficiently to implement the range query data structure using arrays and linking node parents with children through indexes. Therefore, in all our implementations, all structures from Table 2 have linear complexity for construction operation and storage space.

As shown in [22], search, insert and delete operations for segment tree have logarithmic time. The search operation of the KI tree queries the maximum number of vehicles on a segment during a time interval. It queries the tree nodes, starting from root, as follows: if a node is fully included in the queried time interval, its corresponding value is returned; in the case that a node partially intersects the queried time interval, its children are queried recursively. The recursive approach goes top-down in the tree and introduces the worst case complexity factor of $\log_{k}n$ (that represents the height of the tree). Passing all the children of a node takes O(k) and therefore, the search operation complexity is O($k\times \log_{k}n$).

Insert operation sets the time stamp of the time interval when a vehicle enters on a segment. Delete operation sets the time stamp of the time interval when a vehicle leaves a segment. For a vehicle that stays on a segment for a specific time interval, the insert and delete operations on the KI tree behaves as follows: following a bottom-up approach, if a time interval fully covers all the children of a parent tree node, only the parent tree node is considered for changes, otherwise the child nodes information is changed. The information changed in both cases must be propagated bottom-up until it reaches the root of the tree. The number of changes that represent a vehicle that stays on a segment for a specific time interval corresponds to $O(\log_{k}n)$ operations in a KI tree. For each change, it is required to do $O(\log_{k}n)$ steps in order to propagate the change to the root. Additionally, the number of children that are passed in a KI tree for a vehicle that stays on a segment for a specific time interval is O(k). Hence, we have the time complexity for insert and delete O($k\times \log_{k}n+\log_{k}^{2}n$). For the k-ary entry point tree, the search operation has the same flow as for KI tree and therefore the time complexity is O($k\times \log_{k}n$). The KEP tree keeps simpler information than KI tree and therefore, the insert and delete operations are simpler. The insert operation updates the data in all the nodes that logically contain the time represented by the moment when a vehicle enters on a segment. Delete operation updates the data in all the nodes that logically contain the time represented by the moment when a vehicle leaves a segment. In this way, the complexity of both insert and delete operations is O($\log_{k}n$).

A promising range query data structure that we are currently under work for experimental evaluation is Van Emde Boas Tree that has O(log log n) complexity for search, insert and delete operations.

## 5. Algorithms

To answer Q2 and Q3 questions we designed and implemented an algorithm for query and update operations on both KI and KEP trees. In this section we describe the proposed algorithms. For simplicity, in the rest of the paper we denote with Query algorithm the Search operation a range query tree and with Update algorithm the combination of Insert and Delete operations that correspond to the update of a range query tree based on vehicle’s time interval on a segment.

For efficiency reasons, the implementation of the KI tree and KEP tree algorithms (Query and Update) is based on indexed arrays that easily support direct access on the tree nodes. From experimental evaluation from Section 6, we found 10-ary trees (k = 10) to be the best choice. However, for the sake of exemplification, for the below algorithms we use examples with k = 4. We use 1 based indexed arrays to store the K-ary trees, meaning that in this case the time starts at second t = 1. The k-ary tree levels are numbered increasingly from top-down.

In our Query and Update Algorithms for both KI tree and KEP tree we defined the main parameters as follows:

- $t_{\mathrm{start}}$—start of a time interval
- $t_{\mathrm{end}}$—end of a time interval
- l—level of a node in a K-ary Tree
- t—specific time
- $l_{\mathrm{lowest}}$—the lowest level in a K-ary Tree
- ${\mathrm{size}}_{l}$—time interval length corresponding to a node at level l in a K-ary Tree
- $l_{\max}$—maximum level of a K-ary Tree (the lowest level)
- ${\mathrm{mv}}_{l,i}$—maximum number of vehicles at a KI Tree node indexed by level l and position i
- ${\mathrm{llm}}_{l,i}$—maximum number of vehicles from a lower level corresponding to KI Tree node at l and position i
- vpo—vehicles passing over a time interval
- ${\mathrm{vpo}}_{l,i}$—vehicles passing over that corresponds to the K-ary Intreval Tree node at l and position i. ${\mathrm{vpo}}_{l,i}={\mathrm{mv}}_{l,i}-{\mathrm{llm}}_{l,i}$
- start—start index in a KI Tree array storage corresponding to the beginning of a time interval
- end—end index in a KI Tree array storage corresponding to the end of a time interval
- $t_{\mathrm{query}}$—specific time for a query representing an index in the KEP Tree
- ${\mathrm{count}}_{l,i}$—number of vehicles for at a KEP Tree node indexed by level l and position i
- $\max_{l}$—maximum value found at a certain level in a KI Tree. It is passed as a parameter to Propagate routine. Based on its value, the llm at higher levels is changed or not.

### 5.1. K-ary Interval Tree

The KI Tree Query algorithm answers Q2. The pseudo-code of the algorithm is shown in Algorithm  3. In the following paragraphs we describe the implementation of the KI Tree Query algorithm using an example that shows vehicles presence on a segment during time.

**Figure 6:**
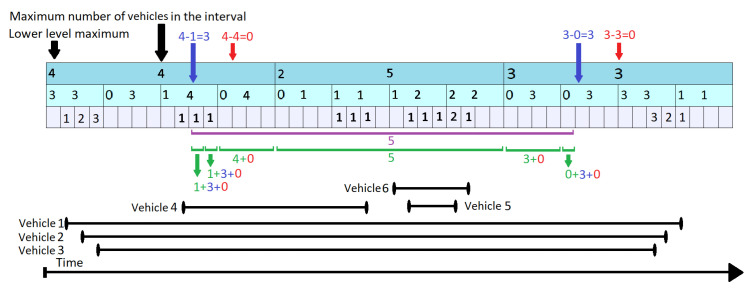
KI Tree Query on Time Interval Starting at Second 11 and Ending at Second 37.

Assuming the requirement to query maximum number of vehicles during time interval given by $t_{\mathrm{start}}=11,t_{\mathrm{end}}=27$, on the segment represented in Figure 6. The queried time interval can be seen in Figure 6 as the area underlined with purple. The green lines in Figure 6 cover the positions iterated by the query, with one green line for each position.

Each gray slot marks a single unit of time, while the above blue layers represent a summary for a larger interval. For our example, we use k = 4 as the ratio between layers. For each layer we keep the maximum number of vehicles which are simultaneously on the segment at some point during the covered interval (${\mathrm{mv}}_{l,i}$—the number in the center of the rectangles) and the maximum number of cars counted by the layers beneath ( ${\mathrm{llm}}_{l,i}$—left corner of the rectangle). The difference between the two numbers gives the number of vehicles passing over (vpo). The black segments from the bottom of the Figure 6 represent the time period when a vehicle is on the segment.
**Algorithm 3** KaryIntervalTreeQuery1:**procedure**Query(*$t_{start}$, $t_{end}$, l, vpo*)2:    $start\leftarrow \mathrm{getIndex}(t_{start},l)$3:    $end\leftarrow \mathrm{getIndex}(t_{end},l)$4:    max←05:    **for** i←start;i<=end;i=i+1 **do**6:        **if** $l=l_{lowest}\mathrm{or}\mathrm{Covers}\left(l,i,t_{start},t_{end}\right)$ **then**7:           **if** $max<mv_{l,i}$ **then**8:               $max\leftarrow mv_{l,i}$9:           **end if**10:        **else**11:           $t_{newStart}\leftarrow i\times size_{l}$12:           **if** $t_{start}>t_{newStart}$ **then**13:               $t_{newStart}\leftarrow t_{start}$14:           **end if**15:           $t_{newEnd}\leftarrow \left(i+1\right)\times size_{l}-size_{l_{max}-1}$16:           **if** $t_{end}<t_{newEnd}$ **then**17:               $t_{newEnd}\leftarrow t_{end}$18:           **end if**19:           $vpo_{new}=mv_{l,i}-llm_{l,v}+vpo$20:           $max_{new}\leftarrow \mathrm{Query}\left(t_{newStart},t_{newEnd},l+1,vpo_{new}\right)$21:           **if** $max<max_{new}$ **then**22:               $max\leftarrow max_{new}$23:           **end if**24:        **end if**25:    **end for**26:    returnmax+vpo27:**end procedure**

The KI Tree Query algorithm runs in a top-down approach starting at topmost level (level 1) and iterating through all the elements that intersect the input interval in at least one point. For the given example, it checks all the three positions at the first level. Since the interval represented at position 1 (first cell in the indexed array from level 1) does not overlap completely over the purple area, it goes to the lower layers until it encounters an interval completely contained in the input. As it descends, it adds up all the passing over vehicles from the superior layers. In Figure 6 it can be seen that the number of vehicles passing over for levels 1 (marked with red) and 2 (marked with blue) are added to the values found at the lowest layer. At time t = 13 the query is back at level 2. It does not need to look at a lower level, as ${\mathrm{mv}}_{2,4}$ overlaps completely the input time interval. Starting with t = 17, the topmost level (level 1) can answer for the next time interval that starts at second 17 and ends at second 32. As we approach the end of the input interval, the query descends again to lower layer and as it goes along it maintains a maximum of all the values found. These values are written down below the green lines. The maximum number of vehicles which are on the segment in the requested interval is 5, given by ${\mathrm{mv}}_{1,2}$.

It is worth mentioning that the KI Tree Query algorithm uses an additional routine called Covers. It decides whether an element in the array identified by the level and position (a KI tree node) overlaps completely a given time interval.

The purpose of the KI Update algorithm, represented by the pseudo-code routines Algorithms 4 and 5, is to update the KI Tree Data that corresponds to a vehicle which passes a specific segment during time interval $\left[t_{\mathrm{start}},t_{\mathrm{end}}\right]$. As for KI Query algorithm, we used the example approach to explain the algorithm’s implementation.
**Algorithm 4** KaryIntervalTreeUpdate1:**procedure**update(*$t_{start}$, $t_{end}$*)2:    $max_{l}\leftarrow 0$3:    $l\leftarrow l_{max}$4:    $t\leftarrow t_{start}$5:    i←16:    **while** $t\leq t_{end}$ **do**7:        **if** $\mathrm{CanGoUp}(t,l,t_{end})$ **then**8:           $\mathrm{Propagate}(l-1,max_{l},t-size_{l})$9:           l←l−110:           $l_{max}\leftarrow 0$11:           continue12:        **end if**13:        **if** $\mathrm{MustGoDown}(t,l,t_{end})$ **then**14:           $\mathrm{Propagate}(l+1,max_{l},t-size_{l})$15:           l←l−116:           $l_{max}\leftarrow 0$17:           continue18:        **end if**19:        i←getIndex(t,l)20:        $mv_{l,i}\leftarrow mv_{l,i}+1$21:        **if** $mv_{l,i}>l_{max}$ **then**22:           $l_{max}\leftarrow mv_{l,i}$23:        **end if**24:        **if** $t\geq t_{end}$ **then**25:           $\mathrm{Propagate}(l-1,l_{max},t_{end})$26:        **end if**27:        $t\leftarrow t+size_{l}$28:    **end while**29:**end procedure**

**Algorithm 5** Propagate
1:**procedure**Propagate(*l, $l_{max}$, t*)2:    **if** l<1 **then**3:        return4:    **end if**5:    i←getIndex(t,l)6:    **if** $llm_{l,i}<l_{max}$ **then**7:        $vpo\leftarrow mv_{l,i}-llm_{l,i}$8:        $llm_{l,i}=l_{max}$9:        $mv_{l,i}=l_{max}+vpo$10:        $\mathrm{Propagate}(l-1,mv_{l,i},t)$11:    **end if**12:
**end procedure**



**Figure 7:**
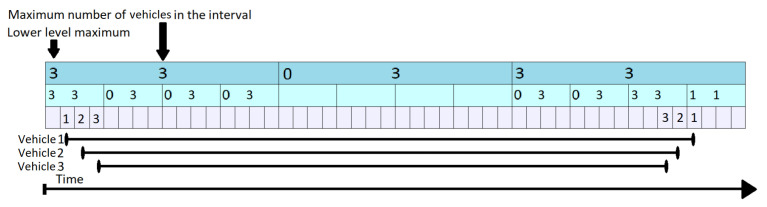
KI Tree—Initial State.

Let us consider that the initial content of the KI Tree is the one shown in Figure 7 where k = 4 and, as for KI Tree Query, each element at the lowest level represents one second.

**Figure 8:**
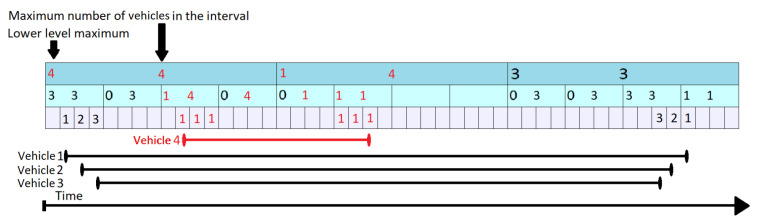
KI Tree Update for Vehicle 4.

In Figure 8, the 4th vehicle wants to enter the segment at time $t_{\mathrm{start}}=10$ and leave at time $t_{\mathrm{end}}=23$ (represented by the red segment on the bottom of the figure). Normally, if there was only the lowest level (level 3), it would have to increment all the values from 10 to 23 one by one. This is not the case, as the values from 13 to 20 can be all increased in two steps. The update procedure is done in a bottom-up manner. At every step, it is checked whether the current position is the first one in an interval covered by the level above. In our case, t = 10 is not, so vehicle number 4 stays on the lowest level and increments the values found at positions 10, 11 and 12 by 1. When it reaches index 13, this corresponds to the beginning of a new interval at level 2. Moreover, this interval ends before time $t_{\mathrm{end}}=23$ when vehicle 4 leaves the segment. As a result, it is allowed to go one level up. Before it does so, the Propagate routine (presented in Algorithm 5), is triggered from Algorithm  4 for the previously covered interval. As some values at a lower level have changed, it is possible that the upper values are no longer valid. By increasing the elements at position 10, 11, 12, the lower level maximum at level 2 position 3, was also increased by 1 (${\mathrm{cpo}}_{2,3}$ was 3). The maximum numbers of vehicles in the interval changes to ${\mathrm{mv}}_{2,3}={\mathrm{llm}}_{2,3}+{\mathrm{cpo}}_{2,3}=4$. Additionally, ${\mathrm{llm}}_{1,1}$ is changed to 4, just like ${\mathrm{mv}}_{1,1}$. This example clarifies the difference between llm and mv and the meaning of “vehicles passing over”: a vehicle is passing over an interval (element) if it uses the segment for the full duration of that interval and causes changes that occur only at higher levels of the KI Tree. Nevertheless, even if we do not update each value from lower levels during that interval, when performing a query we do have to account for that vehicle as well, as a the vehicle also uses the segment during that time.

Returning to our example, when vehicle 4 reaches position 5 at level 2, this corresponds to an interval beginning at the topmost level (level 1). However, the top element goes beyond $t_{\mathrm{end}}=23$, so vehicle 4 stays at level 2. It is allowed to stay at this level until it reaches position 6 (in level 2) and then it has to go one level lower, as position 6 covers an interval ending at t = 24. Before it moves to the lower level, the Propagate routine is triggered again, causing changes at both the first and second layer. Finally vehicle 4 updates elements 21, 22 and 23. The Propagate routine is triggered, but it only has an impact on lower 2 where it sets ${\mathrm{llm}}_{2,6}$ and ${\mathrm{mv}}_{2,6}$ to 1. The value of ${\mathrm{llm}}_{1,2}$ was previously equal to 1 and therefore, no changes are triggered here.

**Figure 9:**
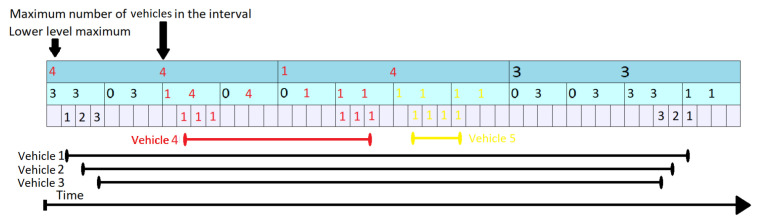
KI Tree Update for Vehicle 5.

Figure 9 shows a 5th vehicle arriving on the same segment at $t_{\mathrm{start}}=26$ and leaving at $t_{\mathrm{end}}=30$ (represented by the yellow segment at the bottom of the figure). This scenario has many similarities with the previous one. The difference from the previous scenario is that the vehicle 5 only affects (increases) values at the lowest level, as it does not stay on the segment long enough to be able to change any of the higher levels. It only triggers changes at layer 2 through the Propagate routine. The topmost level is not modified at all as ${\mathrm{llm}}_{1,2}$ remains 1 even after the arrival of vehicle 5.

**Figure 10:**
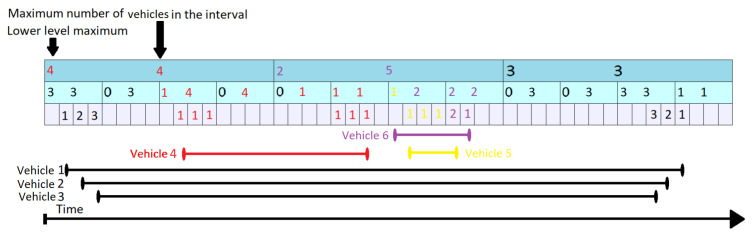
KI Tree Update for Vehicle 6.

In the last scenario of KI Tree Update, shown in Figure 10, we have a 6th vehicle entering the segment at $t_{\mathrm{start}}=25$ and leaving it at $t_{\mathrm{end}}=30$ (represented by the purple line. This corresponds to a time interval started at a level 2 position. In this case the update routine starts execution at level 2 and only goes down to level 3 to update time moments 29 and 30. In this case ${\mathrm{llm}}_{2,7}$ remains unchanged, while ${\mathrm{mv}}_{2,7}$ is increased by 1. Again, the Propagate routine is triggered causing ${\mathrm{mv}}_{1,2}$ to become 5 and ${\mathrm{llm}}_{1,2}$ to become 2.

Besides Propagate, there are a few more routines used by KI Tree Update algorithm, as follows:

- GetIndex is a function that computes the index for a specific time moment at the given interval
- CanGoUp is a function that determines if a data propagation can be done on a higher level (depending on the current time, current level and vehicle time interval on a segment)
- MustGoDown is a function that determines if a data propagation has to be done on a lower level (depending on the current time, current level and vehicle time interval on a segment) the vehicle exits the segment.

### 5.2. K-ary Entry Point Tree

**Figure 11:**
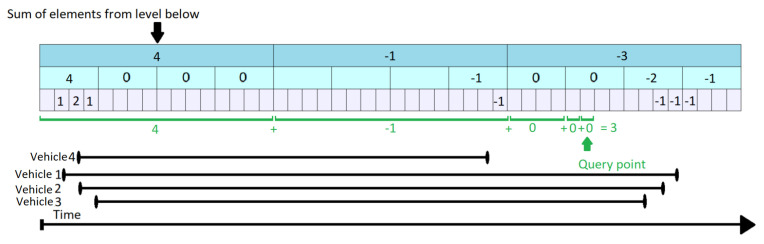
KEP Tree Query at Time t = 38.

The KEP Query algorithm shown in the pseudo-code of Algorithm  6 answers Q3. Because the KEP Tree structure its simpler that KI Tree structure, the implementation of the algorithm is also simpler in comparison with KI Tree Query algorithm. KEP Tree does not need to store lower level maximum (llm) value and therefore, less memory is used by the implementation. As is shown in Figure 11, for each KI Tree node we only have a data field (sum) that aggregates the data from children nodes. Entry Point Tree Query algorithm is described in the next lines using the example in Figure 11. Similar to the KI Tree figures, the lowest, gray level represents a single unit of time while the above, blue layers summarize a larger time frame. For each of the elements we only store a simple vehicles counter (the number in the center of the rectangles). The black segments from the bottom of the figure represent the time interval when a vehicle is the segment. A query at time t is performed by adding all the values from moment 1 up to and including t. It starts at the highest level and, as it becomes closer to t, the query descends to lower levels, so that it does not exceed the queried time interval. The entire queried interval is underlined with green lines in Figure 11 and represents the values of (${\mathrm{count}}_{1,2},{\mathrm{count}}_{2,9},{\mathrm{count}}_{3,37}$ and ${\mathrm{count}}_{3,38}$). These values are summed to obtain the result 3 for t = 38. The lowest level is treated separately, as it has to include the last index as well, whereas the upper levels stop right before it.
**Algorithm 6** KaryEntryTreePointQuery1:**procedure**Query(*t*)2:    result←03:    $t_{query}\leftarrow 1$4:    **for** $l\leftarrow 1;l<l_{max};l\leftarrow l+1$ **do**5:        $start\leftarrow \mathrm{getIndex}(t_{query},l)$6:        end←getIndex(t,l)7:        **for** i←start;i<end;i←i+1 **do**8:           $t_{query}\leftarrow t_{query}+size_{l}$9:           $result\leftarrow result+count_{l,i}$10:        **end for**11:    **end for**12:    $start\leftarrow \mathrm{getIndex}(t_{query},l_{max})$13:    $end\leftarrow \mathrm{getIndex}(t,l_{max})$14:    **for** i←start;i≤end;i←i+1 **do**15:        $t_{query}\leftarrow t_{query}+size_{l}$16:        $result\leftarrow result+count_{l,i}$17:    **end for**18:    returnresult19:**end procedure**

**Figure 12:**
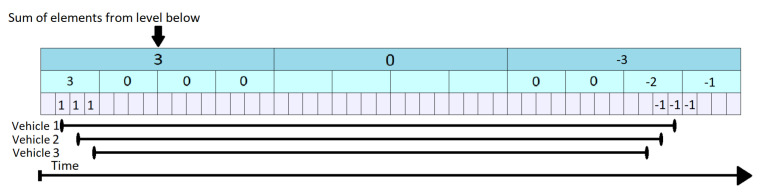
KEP Tree—Initial State.

**Figure 13:**
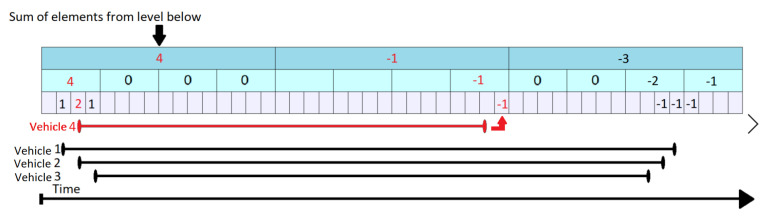
KEP Tree Update for Vehicle 4.

The KEP Update operation is described in Algorithm 7. For any specific segment, it increments by 1 the count value when a vehicle enters on the segment ($t_{\mathrm{start}}$) and decrements by 1 the very next moment after it leaves ($t_{\mathrm{end}}-1$). The time stamp when a vehicle leaves a segment is decreased by one. The increase/decrease operation is done at all the levels which overlap with the given point in time. All other values remain unchanged. Starting from the state in Figure 12, let us suppose that the vehicle 4 enters the segment at $t_{\mathrm{start}}=3$ and leaves the segment at $t_{\mathrm{end}}=31$. In Figure 13 shows the top-down flow of the update operation. It increments the data corresponding to positions that overlap the interval in all corresponding levels (marked by red). This corresponds to ${\mathrm{count}}_{1,1},{\mathrm{count}}_{2,1}$ and ${\mathrm{count}}_{3,3}$. When the vehicle leaves the segment ($t_{\mathrm{end}}=31$), it triggers a decrease operation by 1 at t = 32. Consequently, the values found at ${\mathrm{count}}_{1,2},{\mathrm{count}}_{2,8}$ and ${\mathrm{count}}_{3,32}$ are decreased by 1.
**Algorithm 7** KaryEntryPointTreeUpdate1:**procedure**update(*$t_{start}$, $t_{end}$*)2:    $t_{end}\leftarrow t_{end}+1$3:    **for** $l\leftarrow 1;l\leq l_{max};l\leftarrow l+1$ **do**4:        $start\leftarrow \mathrm{getIndex}(t_{start},l)$5:        $end\leftarrow \mathrm{getIndex}(t_{end},l)$6:        $count_{l,start}\leftarrow count_{l,start}+1$7:        $count_{l,end}\leftarrow count_{l,end}-1$8:    **end for**9:**end procedure**

## 6. Model Evaluation

### 6.1. Evaluation Scenario

**Figure 14:**
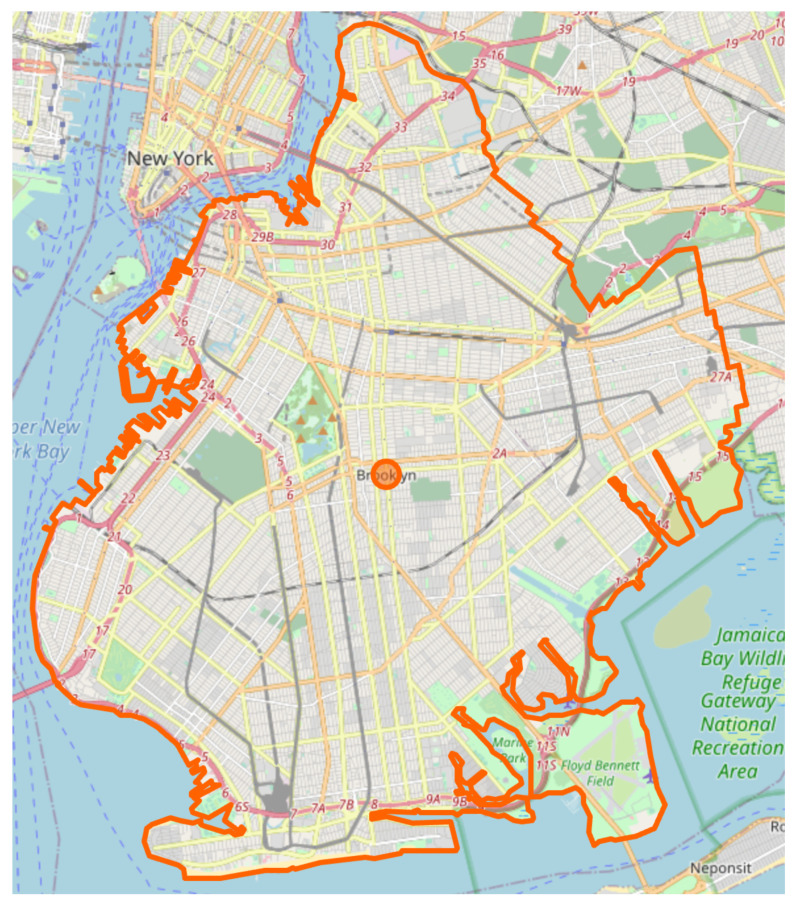
Open Street Map for Brooklyn, New York, USA.

Our model evaluation was done by simulating a large amount of vehicles that generate traffic congestion in an urban area as follows:

- random routes were generated in Brooklyn, New York area shown in Figure 14 (start and end points randomly chosen)
- the random routes represent 10,000 concurrent vehicles that run in a short period of time (10 min) in order to generate traffic congestion

During evaluation we compared three range query data structures (Segment Tree proposed in [22] and our proposed KI and KEP Tree) in terms of performance and their usage impact on simulated traffic congestion scenario. All the results presented below were obtained from tests that run on a Windows computer with an i7 4720HQ processor and 8 GB of RAM (with swap memory extension). OSMAnd ran on this computer as a cloud service that simulates a V2C environment.

### 6.2. Measurements

#### 6.2.1. Metrics

To assess the utility, performance and scalability of the proposed range query data structures and their corresponding algorithms, the following metrics are evaluated and discussed:

- Average Query Time—The average time for executing Query algorithm on a segment
- Average Update Time—The average time for executing Update algorithm on a segment
- Total Number of Queries—Total number of requested queries on segments in order to simulate vehicles through route generation requests
- Total Number of Updates—Total number of requested updates on segments in order to simulate vehicles through route generation requests
- Average Estimated Time of Travel (ETT)—Average Estimated Time of Travel for simulated vehicles through route generation requests

#### 6.2.2. Average Query Time

**Figure 15:**
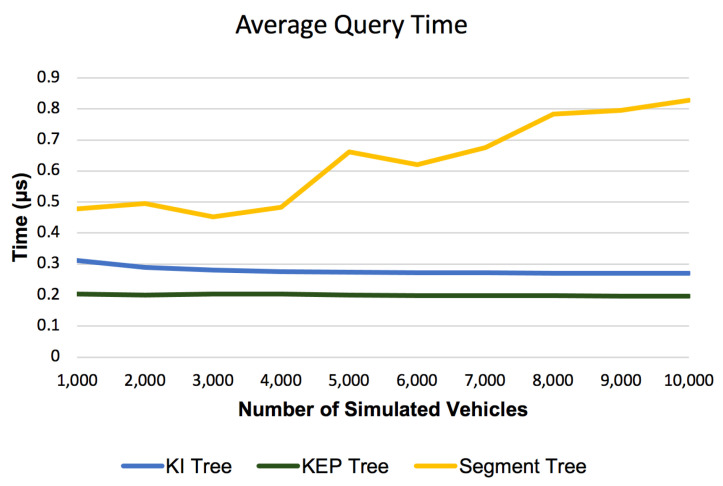
Average Query Time for Segment, KI and KEP Trees.

Figure 15 represents the evolution of the average query time, according to number of simulated vehicles, for the three evaluated range query data structures (Segment Tree, KI Tree and KEP Tree). It can be observed that the Average Query Time for Segment Tree shows a cvasi-linear increase with the number of cars. This is given by the asymptotic complexity of the Segment Tree Search operation from Table 2 and the computational factors of the Segment Tree Search operation implementation. The asymptotic complexity of the Search operations from the data structures we introduced (see Table 2) is strongly affected by the logarithm base (k = 10) allowing query operations of the proposed data structures to maintain a constant run time. In this way, the query operation scales without constraints.

#### 6.2.3. Average Update Time

**Figure 16:**
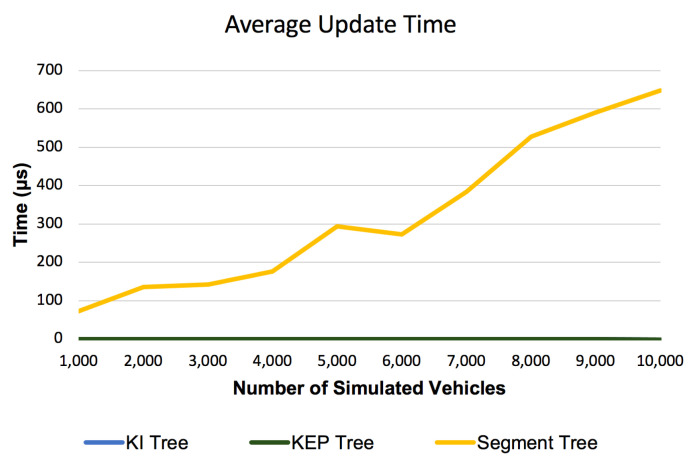
Average Update Time for Segment, KI and KEP Trees.

**Figure 17:**
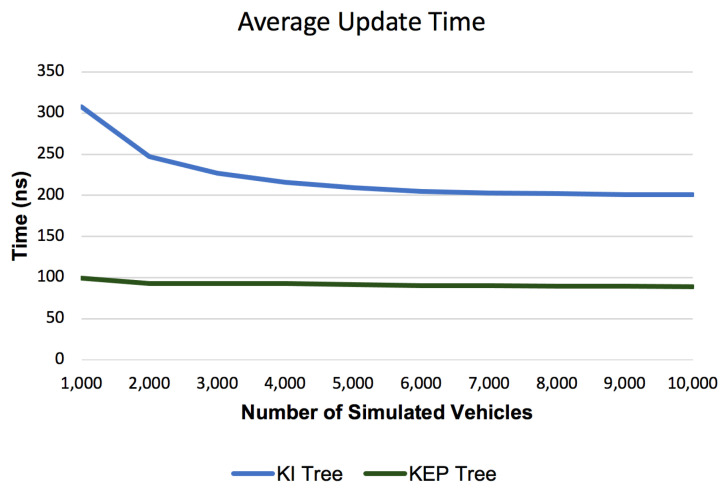
Average Update Time for KI and KEP Trees.

Figure 16 shows the Average Update Time for the proposed data structures compared with segment tree implementation from [22]. It can be observed that segment tree has different size of orders compared with KI tree or KEP tree (microseconds vs. nanoseconds). While the Average Update Time for segment tree increases with the number of simulated vehicles the Average Update Time remains constant at the bottom of the graph and cannot be evaluated. Due to the large gap we need to analyze a separate graph in Figure 17 that contains only Average Update Time for KI tree and KEP tree. The Average Update Time values before having first 3000 simulated vehicles decreases from about 310 nanoseconds to about 200 nanoseconds. This can be interpreted as a spike in the processor usage (by other external running applications) which influences our measurements that are very sensitive due to the measurement scale (nanoseconds). From 3000 simulated vehicles, it can be observed that like the Average Query Time, the Average Update Time also follows a constant pattern.

Like for query operations, the Segment Tree Update operation follows a cvasi-linear pattern given by the asymptotic complexity in Table 2 and the higher implementation factors of that affects the Segment Tree Update operation implementation. The run time for the update operations of the new introduced data structures follows a constant pattern given by the logarithm base (k = 10) from the asymptotic complexities in Table 2 (see. Insert and Delete operations complexities for KI and KEP trees).

#### 6.2.4. Total Number of Queries

**Figure 18:**
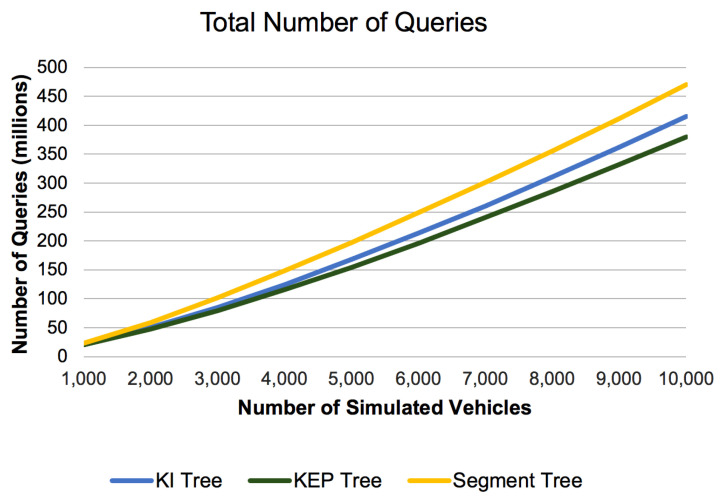
Total Number of Queries Operations.

The graph in Figure 18 shows the total number of queries used to generate routes for vehicle simulation. For the all the range query data structures we tested, the total number of queries grows with the number of simulated vehicles, as expected. It can be observed that the Total Number of Queries for Segment Tree is greater than the Total Number of Queries for our proposed data structures with up to 25%. This happens because they answer different queries and the internal representation of the data structures differs. Specifically, segment trees give more pessimistic results because of the query they answer, thus forcing more alternative routes to be considered and directly increasing the number of queries performed. This is why we measured the average values for query operation as the number of simulated vehicles increases.

#### 6.2.5. Total Number of Updates

**Figure 19:**
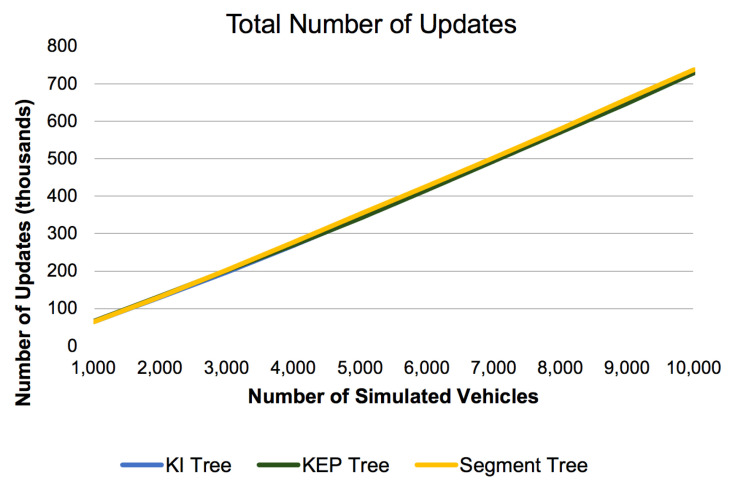
Total Number of Updates Operations.

Figure 19 shows the total number of update requests used for vehicles simulation based on different range query data structures. The values corresponding for the three evaluated data structures are in the same range. The graph exploration algorithm used to generate a route queries many segments (until generates the route) while update requests happen only on the segments that are part of an already generated route. As a consequence, the total number of queries has different order of magnitude compared to the total number of updates (hundred of millions vs. hundred of thousands).

#### 6.2.6. Average Estimated Time of Travel (ETT)

To evaluate the usability and traffic congestion improvement we measured the average ETTs for simulated vehicles that were generated through two different route planning algorithms:

- Basic Routing Algorithm (BRA)—generates individual routes with smallest ETT at the generation moment
- V2C-based Routing Algorithm (V2CRA)—generates routes by targeting congestion avoidance and global time spent in traffic reduction (i.e., reduce average ETT for all the routes). The V2CRA algorithm uses the traffic information stored in range query data structures to improve the average ETT for all routes.

**Figure 20:**
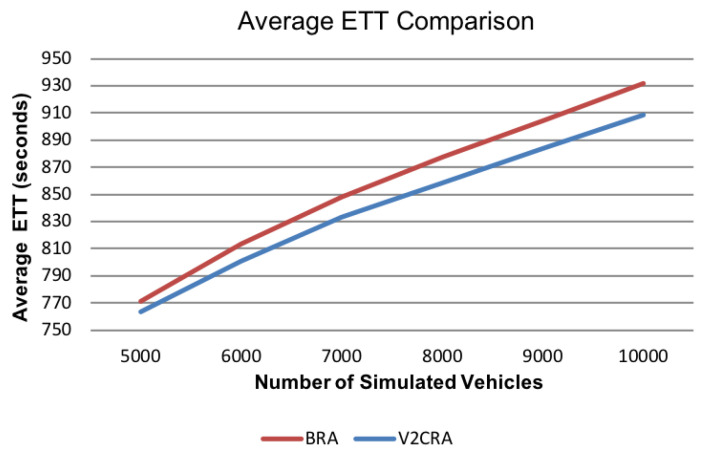
Average ETT for BRA vs. V2CRA.

In Figure 20 we compare Average ETTs for BRA vs. V2CRA algorithms running on Brooklyn, New York. The graph shows the context after 5000 vehicles were simulated and traffic congestion started to appear in the urban area. On average, V2CRA provides routes with 24 s faster than BRA. From this perspective, we can say that our proposed range query data structures can be efficiently used to predict and avoid traffic congestion in urban areas. In this way, the time spent in traffic is reduced and therefore, the urban areas vehicular energy consumption (i.e., pollution) can be reduced by about 2.6%. This percentage can be increased by fine tuning the route planning algorithm’s configuration and is part of future work since the main purpose of this work was to model the urban traffic in an useful, efficient and scalable way.

It is worth mentioning that, like in real life, short segments (e.g., segments in intersections) can become easily congested. Mostly, they are segments that have below 50 meters. We did special treatment for such segments in order to have prediction as accurate as possible.

#### 6.2.7. Scalability

Considering the statistics in [72], an urban scenario with large amount of traffic can have about 100,000 of concurrent vehicles on the roads at peak times. Let’s consider a realistic V2C infrastructure as follows

- computing power equivalent with 1000 parallel computers that we used for our testing.
- 1TB of memory capabilities.

Below is analyzed the output of our measurements:

- 10,000 concurrent vehicles were simulated through route generation
- the total time for query and update operations during route generation is less than 150 s for KI Tree, meaning an average of less than 15 ms for each vehicle. KEP Tree is more than 1.5 times faster thank KI Tree.
- for 10,000 generated routes were navigated 2772 different segments from the map and for each segment we represented vehicle’s information for all the seconds in a day (86,400 s) meaning 300 millions of nodes in KI and KEP trees. A KI or KEP tree node used in the worst case 0.1 KB. In total for all 10,000 generated routes we used about 30 GB of memory.

Based on our measurements results and the proposed realistic V2C infrastructure, for 100,000 concurrent simulated vehicles, the proposed data structures will use in total less than 1.5 s on computation (meaning an average of 0.015 ms for each vehicle) while the memory footprint will be 0.3 TB.

Considering all the above, our proposed data structures are scalable to model real urban traffic scenarios and especially to reduce the congestion.

## 7. Conclusions and Future Work

Considering traffic congestion challenges in urban areas, in this paper we proposed a novel traffic prediction and congestion avoidance approach based on traffic data modeling range query data structures. We introduced two new range query data structures (KI tree and KEP tree) that can be used to model vehicles on the road segments. Congestion avoidance is done by adjusting the cost value of a certain segment based on the number of vehicles on that segment at a certain time. By integrating the proposed data structures and adapting the routing algorithm of the OSMAnd navigation solution the paper reports the following achievements:

- Modelled real urban traffic congestion via simulation of large number of vehicles (thousands) in a short amount of time (minutes);
- Prove scalability of the proposed data structures (KI and KEP) in a V2V infrastructure;
- Predict traffic congestion by generating and controlling vehicle routes via OSMAnd as cloud service. Our approach is designed to be as close as possible to real navigation scenarios in a V2C infrastructure;
- Prove the structures can better predict traffic congestion and allow for improved means of traffic avoidance.

We modelled traffic congestion by simulating 10,000 vehicles following their routes in Brooklyn, New York. Employing the proposed data structures traffic information on a map segment can be obtained in less than a millisecond during route planning, while time for route generation is less than 1.5 s which make them effective on V2C infrastructure.

At this stage, an important challenge regarding traffic congestion avoidance in the context of connected vehicles based on a V2C infrastructure is the run-time of the route planning algorithm in a macroscopic simulation context. Therefore, fixing such an issue will let us do more tests in different urban areas in order to widely test and calibrate our congestion avoidance method. In this way the overall time spent in traffic can be reduced even more. From data representation perspective we are currently working on Van Emde Boas Tree following the same methodology as in [24] in order to compare its performance with the current solution.

From a usability perspective, the proposed solution can be integrated in a traffic simulation tool (e.g., SUMO, INTEGRATION) in future. Moreover, such an approach can be integrated and used in a market navigation application (e.g., OSMAnd).

Another worth-mentioning further work is related to unpredictable traffic events that can be considered and analyzed in terms of impact on the route planning, re-routing and traffic flow.

## Figures and Tables

**Table 1:** Large Scale Traffic Simulation Solutions.

Solution	Vehicle Positions	Traffic Simulator	Road Network	Query Performance	Update Performance
Proposed	K-ary Tree	OSMAnd	Brooklyn, New York	O($k\times \log_{k}n$)	O($\log_{k}n$)
VNS [18]	Quad Tree	DIVERT	Porto City	NA	NA
Elbery [19,20]	NA	INTEGRATION	Downtown Los Angeles	NA	NA
Farag [21]	Grid cell	INTEGRATION	Downtown Los Angeles	O(n)	O(1)
Stan [22]	Segment Tree	OSMAnd	Cluj-Napoca City	O(log n)	O(log n)

**Table 2:** Complexities of Range Query Data Structures.

	Previous Solution	Current Solution		Future Potential Solution
**Operation**	**Segment Tree**	**K-ary Interval Tree**	**K-ary Entry Point Tree**	**Van Emde Boas Tree**
Construction	O(n)	O(n)	O(n)	O(n)
Search	O(log n)	O($k\times \log_{k}n$)	O($k\times \log_{k}n$)	O(log log n)
Insert	O(log n)	O($k\times \log_{k}n+\log_{k}^{2}n$)	O($\log_{k}n$)	O(log log n)
Delete	O(log n)	O($k\times \log_{k}n+\log_{k}^{2}n$)	O($\log_{k}n$)	O(log log n)
Space	O(n)	O(n)	O(n)	O(n)

## Data Availability

Not applicable.
